# Anti-cancer effect of dung beetle glycosaminoglycans on melanoma

**DOI:** 10.1186/s12885-018-5202-z

**Published:** 2019-01-05

**Authors:** Mi Young Ahn, Ban Ji Kim, Ha Jeong Kim, Jang Mi Jin, Hyung Joo Yoon, Jae Sam Hwang, Kun-Koo Park

**Affiliations:** 1 Departmrnt of Agricultural Biology, National Academy of Agricultural Science, RDA, 166 Nongsaengmyung-Ro, Iseo-Myun, Wanju-Gun 55365 South Korea; 2 Korean Basic Science Institiute, Ochang, 863-883 South Korea; 3 Pharmacogenechips Inc., Chuncheon, 200-160 South Korea

**Keywords:** Anti-cancer effect, Queen of *B. ignitus*, *Catharsius molossus*, Glycosaminoglycan, Microarray

## Abstract

**Background:**

Dung beetle glycosaminoglycan is known to possess anti-aging activities. However, its anti-cancer mechanisms are not fully elucidated yet. The objective of this study was to evaluate the anti-cancer effect of insect-derived polymer dung beetle glycosaminoglycan (GAG) after intraperitoneally injecting it to melanoma mice induced by B16F10 cells.

**Methods:**

To determine molecular mechanism involved in the anti-cancer effect of dung beetle GAG, its origin N-glycan under 3KD Dalton was assayed for melanoma cell cytotoxicity. Quantitative comparisons of adhesive molecule on extracellular matrix and activities of tissue inhibitor of metalloprotease 2 (TIMP-2) were also investigated. In vivo anti-cancer effect of dung beetle GAG on solid tumor size, survival time and gene-expression profiles was also assayed using B10F10 melanoma mice model. Mice with induced melanoma were then treated with *Catharsius molossus* (dung beetle) GAG (CaG) at 5 mg/kg for 8 weeks to investigate its anti-cancer effects compared to bumblebee (*Bombus ignitus*) queen glycosaminoglycan (IQG) and *Huechys sanguinea* glycosaminoglycan (HEG).

**Results:**

These N-glycans derived from these GAG were composed of many linear heparinoid polysaccharides, polymers with hexose and N-acetylhexose. Adminstration with these GAGs increased survival time and decreased melanoma sizes in mice, in accordance with their inhibitory effects on cell growth ratio of melanoma B16F10. In addition, treatment with N-glycans derived from theses glycosaminoglycan increased activities of TIMP-2 in HMVEC cells pretreated with TNF-alpha and in melanoma cells, suggesting that they had anti-inflammatory and anticancer activities. In DNA microarray results, compared to control, CaG treated mouse group showed upregulation of 192 genes including collagen,typeI,alpha1 (Col1a1), consistent with the highly increased in vitro extracellular matrix (ECM) adhesion on collagen 1 and up-regulation of heparanase (Hpse). After treatment with CaG, a total of 152 genes were down-regulated, including nuclear RNA export factor (Nxf3) and hyaluronan proteoglycan link protein1 (Hapln1).

**Conclusions:**

Glycosaminoglycan, CaG can strengthen ECM by increasing activity of TIMP-2 and adhesion activity on collagen known to inhibit changes of ECM, leading to tumor cell invasion and progression.

**Electronic supplementary material:**

The online version of this article (10.1186/s12885-018-5202-z) contains supplementary material, which is available to authorized users.

## Background

Glycosaminoglycans (GAGs) are linear mucopolysaccharides with repeating disaccharide units. Although they are minor components of native tissues, they play key roles in regulation of various physiological processes [1]. Many studies have shown that glycosaminoglycans can prevent cancer [2, 3]. Heparan sulfate glycosaminoglycans derived from endothelial cells can selectively inhibit tumor angiogenesis [4, 5]. GAGs also exert anti-cancer effects by altering structures of various tumor cells through sulfated modifications, including chondroitin sulfate (CS) and dermatan sulfate (DS). Hence, altering expression of CS and heparan sulfate (HS) on surface of tumor cells is a potential molecular strategy to prevent malignant transformation and tumor metastasis [2]. Targeting this glycosaminoglycan complex biomolecules might be a novel therapeutic strategy to treat disorders such as cancers, neurodegenerative diseases, and infections associated with receptor for advanced glycation end-products (RAGE) containing disaccharide units (CS-E) [2].

Generally, anticancer activities of insect extract containing GAG are associated with modulation of the production of various cytokines (vascular endothelial growth factor, prostaglandin E_2_, secretary phospholipase A_2_), nitric oxide related to regulation of inflammation, vascular cell adhesion molecule (VCAM), intercellular adhesion molecule (ICAM) related to defense and repair, and matrix metalloproteinases (MMP) in vitro [3]. However, in vivo studies about the effect of insect GAGs on cancer using animal models are limited. Moreover, at molecular level, each heparan sulfate proteoglycan (HSPG) consists of a core protein linked to one or more linear heparan sulfate (HS) chains composed of alternating D-glucosamine and uronic acids, leading to *N*- or *O*-sulfated glycan [5]. Glycosylation reactions of different glycosyltransferases can form complex sugar chains of secreted or cell-surface glycoprotein (*N*-glycans and *O*-glycans) that can be added at asparagine residues (*N*-linked) or at serine and threonine residues (*O*-linked) [6]. Changes in glycosylation, one of the most common protein posttranslational modifications, are considered as hallmarks of cancer. Diverse N-glycans with the complex sugar chain showing diversity can modulate cell migration, cell-cell adhesion, cell signaling, growth and metastasis of cancer as biomarker targets [7]. However, anti-cancer effects of versatile insect GAGs or N-glycans (purified from theses GAGs) on melanoma using animal models are insufficient.

It has been reported that dung beetle (*Catharsius molossus*, Ca) GAG possesses marked anti-aging activity by reducing oxidative damage and hepato-cellular biomarker levels in aged rats [8]. As an apicultural product, bumblebee (*Bombus ignitus*) queen (IQG) glycosaminoglycan has potential efficacy for treating obesity in high-fat diet rats [9] because it can significantly decrease cellular oxidative damage by decreasing lipid malondialdehyde and protein carbonyl contents. Another insect glycosaminoglycan (HEG) derived from black and scarlet cicada (*Huechys sanguinea*) has been used as an insect crude drug for amenorrhoea, scrofula, and scabies [10]. In this study, we found that glycosaminoglycan from dung beetle and IQG displayed anti-cancer properties. These compounds might hold great promises as anti-cancer agents. We also determined the potential of *C. molossus* GAG to lessen activities of tissue inhibitor metalloprotease (TIMP-2), an inflammation marker [11], in HMVEC ells and melanoma cells. In addition, serum parameters and gene expression levels in B16 F10 induced C57BL/6 N mice after treatment with GAGs were determined.

Our results revealed that GAGs from dung beetle (CaG), IQG, and HEG displayed anti-cancer properties in melanoma cells and melanoma mice. Thus, these GAGs and their dis-composited N-glycans by endoglycosidase F might hold great promises for use as anti-cancer agents. Our results also demonstrated that CaG and IQG could decrease deleterious aspects of cancer in serum, extend survival time, and decrease melanoma sizes in mice by regulating gene expression levels in B16F10 induced melanoma mice after treatment for 6 weeks.

## Methods

### Preparation of insect glycosaminoglycan

Dried *C. molossus and H. sanguinea*, were purchased at a local market in China. Bumble bee (*B. ignitus*) queen was reared and freeze-dried, in the Department of Agricultural Biology, National Academy of Agricultural Science, South Korea. Chondroitin sulfate was purchased from Sigma Aldrich (St. Louis, MO., USA).

Each crude glycosaminoglycan was purified from dried insect (1 kg each) according to previously reported procedures [12]. Dialyzed crude GAG was freeze-dried to obtain concentration up to 0.9%. Crude GAG was loaded onto a DEAE Sephadex A-25 gel chromatography column (40 × 1.2 cm) equilibrated with 50 mM phosphate buffer (pH 7.4). Fractions were eluted using a linear sodium chloride gradient (0 to 2.5 M NaCl in phosphate buffer) at a flow rate of 20 ml/h. Dialyzed glycan was freeze-dried to obtain pure GAG. From each dried insect, the yield of freeze-dried GAG was about 1.52 g for CaG, 2.8 g for IQG, and 8.8 g for HEG as powder. To determine their purities, fractionation of these digested GAGs, and oligosaccharides mixture by GAG enzymes (heparinase I, II, III, etc.) by strong anion exchange (SAX) high performance liquid chromatography with SAX column (Phenomenex, 5 μm, 250 × 10.0 mm, Torrance, CA, USA) afforded oligosaccharides. Liquid chromatography with mass detector (LC-MS, 2020 and AXIMA Resonance, Shimadzu) was sufficient to purify glycosaminoglycans for structural characterization.

### *N*-glycan preparation derived from insect glycosaminoglycan

GAG was purified to obtain low molecular weight *N*-glycan by enzymatic release and enrichment of *N*-glycan via solid-phase extraction. Briefly, each glycosaminoglycan was denatured via rapid treatment at 100 °C for 10 min in an aqueous solution of 100 mM ammonium bicarbonate and 5 mM dithiothreitol. After cooling, 2.0 μL (or 1000 U) of peptide N-glycosidase F (PNGaseF) (New England Biolabs, Ipswich, MA, USA) was added to release N-glycans. The mixture was then incubated in a 37 °C water bath for 16 h. Graphitized carbon cartridges (Extract clean™ carbo, carbograph**,** Grace davision discovery Sciences, Deerfield, IL, USA) were washed with 80% acetonitrile/0.10% trifluoroacetic acid (*v*/v) and conditioned with water. Released *N*-glycans were loaded onto these cartridges and washed with pure water to remove any salt and buffer residues.

### Identification of *N*-glycan derived from CaG, IQG or HEG

Dried *N*-glycans were suspended in 25% acetonitrile/0.05% trifluoroacetic acid in water (v/v) (acidic fraction) and mixed well. They were chromatographed at a condition of 50% acetonitrile eluent with 2,5-dihydroxybenzoic acid (DHB) as a Matrix for MS and MS/MS analysis using Matrix assisted laser ionization (MALDI) Time of flight (TOF) analyzer (AXIMA Resonance, Shimadzu) in positive polarity. Quadrupole ion trap was acquired in a positive ion mode over a mass range of *m/z* 100–2000 at 800~5000 m.

### Anti-cancer activity of *N*-glycan derived from CaG, IQG or HEG in melanoma cells

To determine the anticancer activity of purified N-glycan derived from insect GAG, cytotoxicity against B16F10 melanoma cells was determined, using Cell Proliferation Kit (XTT, sodium 3′-[1-(phenylamino-carbonyl)-3,4-tetrazolium]-bis(4-methoxy-6-nitro) benzene sulfonic acid hydrate) (Roche Diagnostics GmbH, Mannheim, Germany) according to the kit manual. To ascertain cytotoxicity against cancer cells, cell growth ratios of melanoma cells were calculated with respect to sample GAGs using the XTT dye uptake method [3].

### TIMP-2 activity in microvascular endothelial cells and melanoma cells

TIMP-2 activity in human microvascular (cardiac) endothelial cells (D- HMVECs) obtained from type 2 diabetics [Clonetics™, diabetic type II, Lonza CC-2928 (D-HMVEC-C diabetic type II), Cambrex,Walkersville, MD, USA] was measured. TIMP-2 activity in melanoma cultured B16F10 cells was also assayed. Briefly, cells were grown in an endothelial cell basal medium (EBM)-2 with EGM-2 singlequots (Cambrex) at 37 °C in an atmosphere containing 5% CO_2_. Cells pretreated with 0.2 mg/ml of individual N-glycan were incubated for two days prior to determination of TIMP-2 activity using TIMP-2 Immunoassay kit (Quantikine, R&D Systems, Inc., Minneapolis, MN, USA) according to the manufacturer’s instructions as described previously [11]**.**

### Extracellular matrix (ECM) adhesion assay in melanoma cells

Cell adhesion plays a major role in cellular communication and regulation, especially in cancer development [13]. ECM Cell Adhesion Array Kit provides ECM Array microtiter plate with colorimetric detection. It is an efficient method for characterizing cell adhesion. Each well was pre-coated with a different ECM protein (Purified Human Collagen I, II, IV, fibronectin, laminin, tenascin, and vibronectin) along with one BSA-coated well (negative control). For this assay, B16F10 cells (5 × 10^5^ cells) were treated with sample N-glycans (0.2 mg/ml) samples, incubated for two days and assayed using CHEMICON® ECM Cell Adhesion Array Kit (colorimetric, ECM-540, EMD Millipore Co., USA).

### In vivo anticancer study using animal model

C57BL/N6 mice (male) at 8-weeks of age were supplied from Samtako Co. Ltd. (Osan, Korea). All procedures were in accordance with the Korean Ministry of Food and Drug safety (KMED) Guidelines for Care and Use of Laboratory Animals. All animal experiments were approved by Laboratory Animals’ Ethical Committee of the National Academy of Agricultural Science, Rural Development Administration, South Korea (approval number: NIAS 201606). All procedures followed national guidelines for the care and use of animals (individual housing). Mice were acclimated for six months under normal husbandry conditions (temperature, 23 ± 2 °C; humidity, 55 ± 10%; light/dark cycle, 12 h/12 h). They were provided free access to normal diet (D10001, AIN-76A rodent diet, Research Diet Inc., New Brunswick, NJ, USA) and water ad libitum. These mice were allocated into one control group and three treatment groups (13 mice per group). They were distributed according to similarity in weight (22.1 ± 0.5 g). Each treatment was given in PBS daily administered intraperitoneally. The following groups were used: 1) control (B16F10 melanoma, PBS vehicle only, CON), 2) 5 mg/kg CaG (CaG5), 3) 5 mg/kg IQG (IQG5), and 4) 5 mg/kg HEG (HEG5). Mice in each group were maintained with normal diet (AIN-76A rodent diet, Research Diet).

### In vitro melanoma cell culture and cell transfection

B16 F10 murine melanoma cells were routinely cultured in Dulbecco’s modified Eagles medium (DMEM) containing 5% fetal bovine serum (FCS), penicillin, streptomycin and nystatin (complete medium). Upon reaching 90% confluency, cells were harvested using 0.25% trypsin and 0.02% EDTA in PBS. Harvested cells were washed once with complete DMEM containing 5% FCS, and twice in plain DMEM. Cells used in this study were confirmed to have viability of greater than 95% as a single cell suspension. For experimental metastasis assay, 0.1 ml of the suspension (10^4^ cells in 0.2 ml) was injected subcutaneously to each mouse. Animals were sacrificed at 40 days after tumor injection. Tissue and serum were prepared and biochemically assayed. Microscopic examination (hematoxylin and eosin staining) was carried out for histopathological detection of tumor and liver sections.

### Body weight and tumor size detection

Body weights and tumor sizes in each group (*n* = 10) were measured every week. Tumor appeared at approximately the 10th day (2nd week) after implantation of melanoma cells. Therefore, tumor size (cm^3^) was recorded weekly at the largest diameter and height from the 3rd week to the 8th week using a digimatic caliper (Mitutoyo, Co., Japan). To detect survival time, amounts of tumors in each mouse were not determined on a fixed day because mouse because mouse had to be scarified.

### Blood sampling and serum assay

To investigate the effect of treatment on initiation melanoma development, three mice from each group (CON, CaG5, IQG5, and HEG5) were sacrificed for serum assay and DNA microarray at 6 weeks after treatment. Approximately 1 ml of blood was collected from the posterior vena cava under light CO_2_ inhalation and used for serum chemistry measurements. The following parameters were examined: blood levels of carcinoembryonic antigen (CEA), calcitonin, albumin, alkaline phosphatase (ALP), glutamic pyruvic transaminase (GPT, also known as ALT), glutamic oxaloacetic transaminase (GOT, AST), high-sensitivity C-reactive protein (hs-CRP), lactate dehydrogenase (LDH), and total protein. These parameters were evaluated using an autoanalyzer (Hitachi 7060 Automatic Clinical Analyzer, Tokyo, Japan).

### Survival time detection

Survival of mice with melanomas began to be observed at 2 weeks (exactly 10th day) after B16F10 cells injection. Survival time of melanoma mice treated with CaG, IQG or HEG was recorded to ascertain anticancer activity in each group (*n* = 10 in each group) [14].

### RNA preparation and quantitative real-time PCR analysis

Total RNA was isolated from melanoma tissue of melanoma mice treated with sample GAG. Complementary DNA (cDNA) was synthesized from 1 μg of total RNA to according to previously reported procedures [8]. For detection of target gene transcripts, we designed specific forward and reverse oligonucleotide primers using Beacon Designer software (PREMIER Biosoft, Palo Alto, CA, USA). All samples were analyzed in triplicate. Primer sequences for amplification of genes involved in anticancer mechanism and glyceraldehyde 3-phosphate dehydrogenase (GAPDH) internal standard (Table 1).

**Table 1:** Primer sequences for amplication of genes involved in cancer mechanism and GAPDH internal standard

Gene Symbol	Gene Name	Primer Sequence
ACE1	Angiotensin converting enzyme 1	5′-ACAGGCTCCATCCATCCTCC-3’
		5′-TGGGGACAGCAAAGGAGGAA-3’
GAPDH	Glyceraldehyde-3-phosphate dehydrogenase	5’-GTGGAGATTGTTGCCATCAACGA-3’
		5’-CCCATTCTCGGCCTTGACTGT-3’
GPX1	Gluthathione peroxidase 1	5′-CCAACACCCAGTGACGACC-3’
		5′-CTCAAAGTTCCAGGCAATGTC-3′
Hpse	Heparanase	5′-ACTTGAAGGTACCGCCTCCG-3′
		5′-GAAGCTCTGGAACTCGGCAA-3′
Protein tyrosine phosphatase, S	Protein tyrosine phosphatase, receptor type, S	5′-CAAGCTGCCCCTCTCTCTCT-3’
		5′-GCAGAAAGATCACACTGGCCC-3’

### DNA microarray procedure

Microarray hybridization was performed using mouse melanoma samples. Total RNA was isolated from melanoma tissue of melanoma mice using a Qiagen RNeasy Midi Kit (Qiagen, Valencia, CA, USA). A FairPlay™ microarray labeling kit (Stratagene, La Jolla, CA, USA) was used according to the manufacturer’s instructions. Labeled DNA was loaded onto a microarray chip. A hybridization chamber was assembled with the microarray chip of Mouse Gene 2.0 ST Arrays (Affymetrix Inc., Santa Clara, CA, USA) and submerged in a water bath overnight at 60 °C. The microarray chip was washed with wash buffers I, II, and III. The slide was dried by centrifuging and scanned with a BMS Array Scanner (Applied Precision Array WoRx eBiochip Reader (BioRad, Dallas, TX, USA) [15].

### Statistical analysis

Means and standard errors of all parameters were determined for each group using analysis of variance (ANOVA). Student’s *t*-test was used to determine significant differences between control and treated groups. A *p* value < 0.05 was considered statistically significant.

## Results

### Anti-cancer activity of N-glycan derived from CaG, IQG, or HEG

**Fig. 1:**
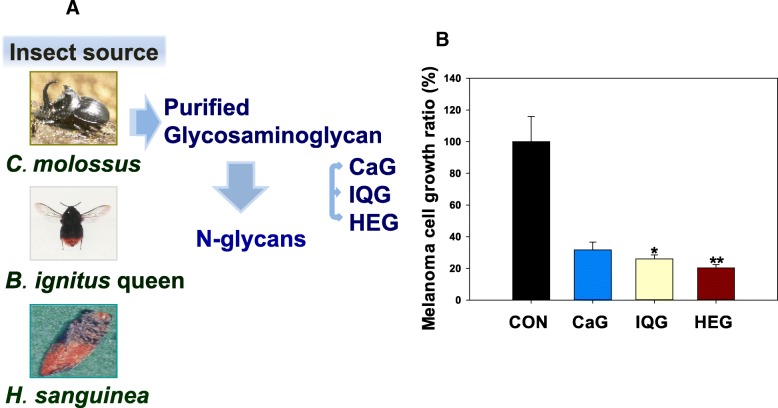
Anti-cancer activity of dung beetle glycosaminoglycan against melanoma cells. **a** Glycosaminoglycans and N-glycans were purified from insect sources: *C. molossus*, *B*. *igitus queen*, and *H. sanguinea*. **b** Measurement of growth ratios of melanoma (B16/F10) cells treated with N-glycans from various insect glycosaminoglycans for two days. CaG: dung beetle (*C. molossus*) glycosaminoglycan (40 μg/ml); IQG: bumblebee (*B. ignitus*) queen glycosaminoglycan (40 μg/ml); HEG: *H. sanguinea* glycosaminoglycan (40 μg/ml). Each value represents mean ± SE. *n* = 3. *p < 0.05, **p < 0.01 significant difference vs. control (PBS) group

From *C. molossus*, *B. ignitus* queen and *H. sanguinea***,** glycosaminoglycan macromolecule polymers, were purified (Fig. 1a). To determine the precise mechanism of action (chemical-structure relationship) in in vitro study, each N-glycan under molecular weight of 3 K Dalton from each GAG, was preferentially purified and assayed for its anticancer activity. Its chemical structure was also characterized. The anti-cancer activity of N-glycan derived from CaG, IQG, or HEG was investigated against cancer (melanoma) cell lines. In XTT assays, each GAG showed cytotoxicity against B16F10 melanoma cells according to decrease of cell growth rate. Cell growth ratios of melanoma cells were significantly decreased by GAG N-glycan samples (40 μg/ml), especially CaG (70.9% decrease, CaG vs CON, *p* < 0.05), IQG (76.2%) and HEQ (81.3%, HEG vs CON, *p* < 0.01) compared control, PBS treated group (Fig. 1b).

### Identification of *N*-glycan derived from CaG, IQG or HEG

**Fig. 2:**
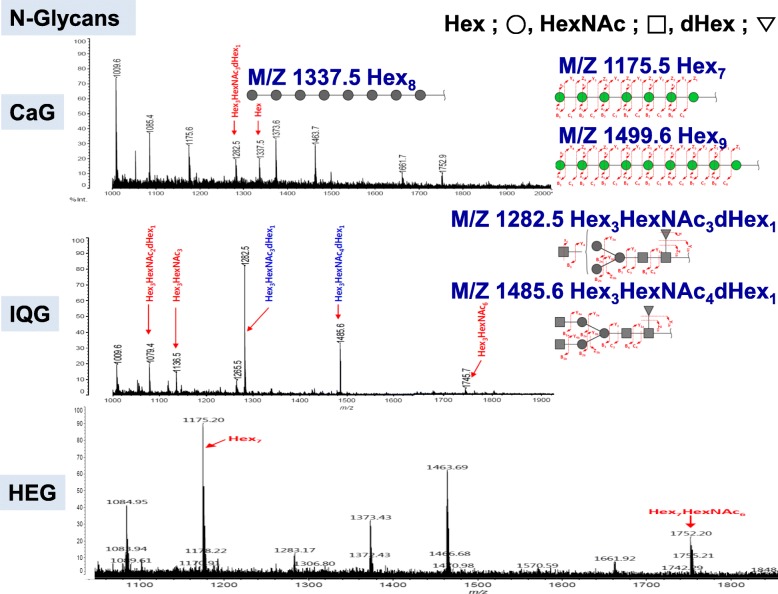
Identification of N-glycans from CaG, IQG, and HEG using MALDI-FOF MS and MS/MS spectrometry. N-glycan of CaG mass (MS) spectrum had main peaks with m/z 1175.5, Hex_7_; m/z 1337.5, Hex_8_; m/z 1499.6, Hex_9_ and m/z 1282.5, Hex_3_HexNAC_3_dHex_1_. N-glycan of IQG had main peaks in MS spectrum with m/z 1282.5, Hex_3_HexNAC_3_dHex_1_; m/z 1485.6, Hex_3_HexNAC_4_dHex_1_ and 1752.2, Hex_7_HexNAC_6_. N-glycan of HEG had main peaks in MS spectrum with m/z 1175.2 Hex7 and m/z 1752.2, Hex_7_HexNAC_6_. Proposed structure was analyzed by N-glycan sequence data library matched with MS/MS spectrum

Glycosaminoglycan is a macromolecule polymer. To determine its precise mechanism of action in an in vitro study, each N-glycan under molecular weight of 3 K Dalton, was preferentially used and characterized. Insect glycosaminoglycans were composed of many linear heparinoid polysaccharides. These insect N-glycans were identified polymer with hexose and N-acetylhexose in this study. Monosaccharide compositions of CaG [8] and IQG [9] have been reported. N-glycan of CaG was identified as Hex _3_ (Hex = arabinose or rhamnose) HexNAc_3_dHex_1_ by MS/MS spectrometry. N-glycan of IQG was also identified as Hex_3_ (Hex = rhamnose) HexNAc_2-4_dHex_1_ or HeX_3_HexNAc_3_ or HeX_3_HexNAc_6_. N-glycan of HEG was identified as a Hex_7_, Hex_7_HexNAc_6_ (Fig. 2, Table 2). N-glycan of HEG composition was Hex_7_, Hex_7_HexNAc_6_. Neutral mono-sugar of HEG was mainly α-fucose based on by trimethylsilane (TMS) gas chromatography-mass data base (data not shown).

**Table 2:** N-glycan mass and mass/mass spectrogram data of CaG, IQG, and HEG

Insect GAG	m/z (logic value)	m/z (detection value)	Intensity (Apex, mV)	Composition			Charges
				Hex	HexNAc	dHex	
CaG	1282.5	1282.5	9.33	3	3	3	+ 1 (Na)
	1337.4	1337.5	9.73	8	0	0	+ 1 (Na)
IQG	1079.4	1079.4	198.41	3	2	1	+ 1 (Na)
	1136.4	1136.5	139.60	3	3	0	+ 1 (Na)
	1282.5	1282.5	817.43	3	3	1	+ 1 (Na)
	1485.5	1485.6	323.55	3	4	1	+ 1 (Na)
	1745.6	1745.7	33.57	3	6	0	+ 1 (Na)
HEG	1175.4	1175.2	128.07	7	0	0	+ 1 (Na)
	1752.6	1752.2	34.56	5	3	2	+ 1 (Na)

*Hex* hexose, *HexNAc* N-acetylhexosamine, *dHex* deoxyhexose

*C. molossus* glycosaminoglycan (CaG) constituted of hexoses in the 3 K Da under fraction, Hex_3_HexNAc_3_dHex_1_ (1282.5 m/z) and Hex_8_ (1337.5 m/z) N-glycan glycosylation structure. IQG N-glycan had the glycosylation structure of the Hex_3_HexNAc_3_dHex_1_ (1282.5 m/z) or Hex_4_HexNAc_3_dHex_1_ (1485.6, m/z). HEG N-glycan had the glycosylated Hex_7_ (1175.2 m/z) and Hex_7_HexNAC_6_ (1752.2 m/z)

### TIMP-2 levels in microvascular endothelial cells and melanoma cells

**Fig. 3:**
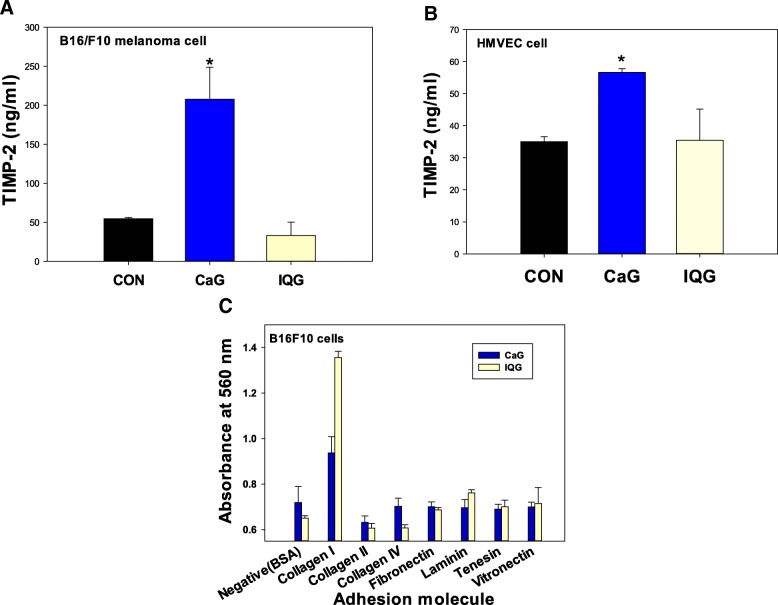
TIMP-2 activity and ECM adhesion. **a** TIMP-2 activity in melanoma cells following treatment with CaG or IQG N-glycan for two days. **b** TIMP-2 activity of CaG or IQG N-glycan in HUVEC cells in pretreated with TNF-alpha. Each value represents mean ± SD. *N* = 3, *p < 0.05 vs CON (PBS) group. TIMP-2, a secreted protein known to prevent degradation of the ECM, was increased by N-glycan from CaG in both melanoma and HMVEC endothelial cells. Otherwise, TIMP-2 level of N-glycan from IQG was selectively decreased in melanoma cells so that ECM of melanoma could be degraded. **c** ECM adhesion activity (microarray) colorimetric detection of N-glycan of CaG or IQG on melanoma cells incubated with various extracellular matrix adhesion molecules: collagen, fibronectin, laminin, tenascin and vibronectin. Both N-glycans from CaG and IQG were found to have high ECM adehesion activity for Collagen I. Each value represents mean ± SD. *N* = 3

Results of TIMP-2 revealed that concentration levels of TIMP-2 in CaG5 treated melanoma cells were higher than those in IQG, HEG, or control groups: CON, 54.69 ± 1.54 ng/ml; CaG5, 207.65 ± 41.18 ng/ml (CaG5 vs. CON, *p* < 0.05); and IQG5, 23.82 ± 30.36 ng/ml (Fig. 3a). TIMP-2 levels in CaG5 treated HMVEC cells were also higher than those in IQG, HEG, and control groups: CON, 35.03 ± 1.54 ng/ml; CaG5, 56.62 ± 1.16 ng/ml (CaG5 vs. CON, *p* < 0.05) ng/ml; IQG5, 35.43 ± 9.75 ng/ml (Fig. 3b).

### ECM adhesion detection in melanoma cells

Results of adhesion of melanoma cells treated with various extracellular matrix (ECM) component proteins, including collagens I, II, and IV, fibronectin, laminin, tenascin, and vibronectin are shown in Fig. 3c. Adhesion ability of IQG treated cells to collagen I was higher than that of CaG-treated cells or other adhesion molecules of ECM.

### Effect of IQG5, CaG5 or HeG5 on the survival of melanoma mice induced with B16F10

**Fig. 4:**
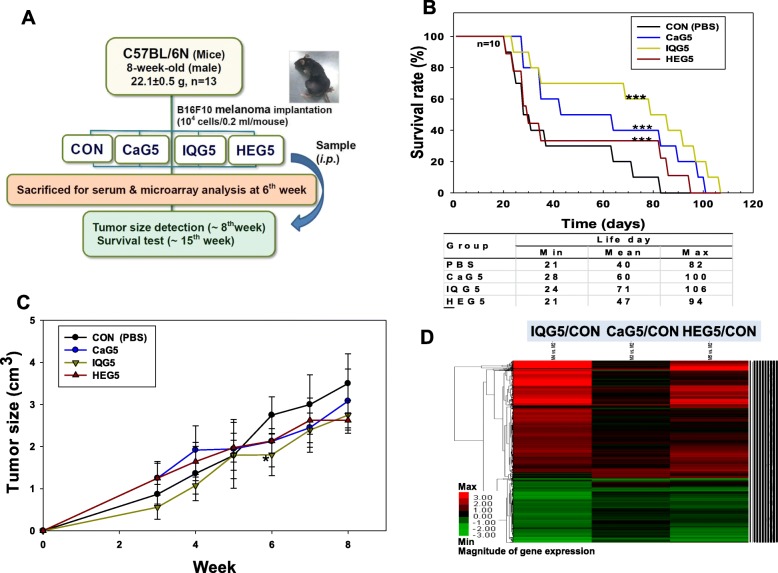
Anti-cancer effect of ding beetle glycosaminoglycan on melanoma mice. **a** Animal experimental design. CaG5: *C. molossus* (dung beetle) glycosaminoglycan 5 mg/kg; IQG5: *B. ignitus* queen glycosaminoglycan 5 mg/kg; HeG5: *H. sanguinea* glycosaminoglycan 5 mg/kg. **b** Survival curve of B16/F10 melanoma mice treated with various insect glycosaminoglycans. *N* = 10, ***p < 0.001 means each sample group significantly different from the control group. **c** Tumor size of B16/F10 melanoma mice treated with various insect glycosaminoglycans from 3 to 8 weeks. *N* = 10, * *p* < 0.05 means each sample group significantly different from control (PBS treated) group by t-test. **d** Heat map of microarray on melanoma tissue of B16F10 melanoma mice treated with CaG5, IQG5 or HEG5

For in vivo study, insect glycosaminoglycans instead of N-glycans were used due to the amount of purified GAG acquired (Fig. 4a). Treatments of melanoma-induced mice with these GAGs resulted in similar lifespan distribution (Fig. 4b). Survival rates of melanoma induced mice treated with insect GAGs were significantly higher than those of control melanoma-induced mice (all sample groups vs non-treated control group, Mann-Whitney Rank Sum test *p* value ≤0.001). Survival rate (%) and mean half-life (days) results are as follows: control mice, 37.66 ± 30 (%) and 40 days; CaG5 group, 55.60 ± 50 (%) and 60 days; IQG5, 66.54 ± 70.0 (%) and 71 days; and HeG5, 43.72 ± 33.3 (%) and 47 days, respectively. IQG5 was founded to have the most positive effect on survival of melanoma mice among insect glycosaminoglycan treatment groups (CaG, IQG, or HEG).

### Body weight and tumor size detection

There were no significant differences in total mean body weight between treatment groups and control groups at the beginning of 8-week treatment: CON, 27.2 ± 4.2 g; CaG5, 27.5 ± 3.8 g; IQG5, 25.4 ± 2.9 g and HeG5, 27.1 ± 3.9 g. Body weights at the end of 8-week treatment were: CON, 31.5 ± 0.5 g; CaG5, 26.8 ± 4.1 g; IQG5, 28.1 ± 2.4 g (IQG5 vs. CON, *p* < 0.05); and HEG5, 29.2 ± 0.8 g (HEG5 vs. CON, *p* < 0.05). Tumor sizes of melanoma-induced mice treated with various insect GAGs according to time (week) are shown in Fig. 4c. The tumor size (cm^3^) of mice treated with CaG, IQG, or HEG was smaller than that in CON (PBS) group. At 6-week, tumor size of IQG or HEG was also significantly smaller than that in CON group: CON, 2.75 ± 0.43 cm^3^; CaG, 2.13 ± 0.61 cm^3^; IQG, 1.80 ± 0.50 cm^3^ (IQG5 vs. CON, *p* < 0.05); and HEG, 2.13 ± 0.30 cm^3^ (HEG5 vs. CON, *p* < 0.05) (Fig. 4c). Melanoma sizes of just dead melanoma mice were also measured to determine amounts of tumors in each mouse during the treatment period. Tumor size was reduced in each mouse treated with GAG, although the reduction was only significant after week 6.

### Sero-biochemical finding of insect GAG in melanoma mice

Previous report has suggested that immune-related enzymes including alkaline phosphatase and LDH are upregulated by chemotherapeutic agents [15]. On the 40th day (6th week) post treatment, sera levels of albumin (g/dL), calcitonin (ρg/ml), CEA (ng/ml), CRP (mg/ml), or LDH (U/L) in melanoma mice were not significantly different between treatment groups and control groups (data not shown). Results of mean ALP levels (U/L) related to immune-antibody were increased as follows: CON, 30; CaG5, 15; IQG5, 49; and HEG5, 60. Mean lactic acid level was also increased in HEG5 groups: CON, 6.0 (U/L); HEG5, 427.5 (U/L).

### Gene expression by quantitative real-time PCR analysis

Some genes were elucidated by real-time PCR. Data are presented as PCR cycle number (Ct value) when concentration of PCR amplicon was equilibrated by sample amplicon. Median CT values of four samples (CON, CaG5, IQG5, and HEG) are presented. Mean Ct value for heparanase in different groups were as follows: CON 27.0, CaG5 25.1(about fourfold increase), IQG5 26.7 and HEG5 26.1 (about twofold increase). For glutathione peroxidase 1 (Gpx1), they were: CON, 27.8; CaG5, 27.5; IQG5, 26.7(about twofold increase); and HEG5, 26.6 (about twofold increase). For protein tyrosine phosphatase S, they were: CON, 26.2; CaG5, 26.4; IQG5, 27.3; and HEG5, 26.3(about twofold decrease). For angiotensin converting enzyme (related cell movement to cancer tissue): CON 33.2; CaG5 33.1, IQG5, 31.0 (about fivefold increase); and HEG5, 32.0 (about twofold increase).

### Gene expression profiling by DNA microarray

To determine gene-expression profiles in CaG, IQG, or HEG-treated B16F10 melanoma-C57BL/6 N mice, microarray analysis was performed using a Mouse 28 K cDNA clone array for each group melanoma sample in order to provide information on potential anti-cancer markers (Fig. 4d). Compared to the control group, CaG5, IQG5, and HEG5 groups exhibited ≥2-fold (200%) increase in expression of 192, 1758, and 1004 genes, respectively. Numbers of up-regulated and down-regulated (in expression level) genes in the IQG5 group were more than those in other groups. In CaG5, IQG5, and HEG5 groups compared to the control group, 152, 1017, and 412 genes were down-regulated ≥2-fold, respectively. These genes were related to transport (*n* = 535), stress response (*n* = 553), cell growth (*n* = 22), and cell division (*n* = 28).

In mice treated with CaG5 compared to the control, 192 genes were upregulated, including collagen, type XII, alpha1 (Col12a1), hemoglobin, beta adult minor chain (Hbb-bt), amino levulinic acid synthase2, erythroid (Alas2), and heparanase (Hpse) (Additional file 1: Supplementary data 1). However, 152 genes were down-regulated, including nuclear RNA export factor (Nxf3), hyaluronan and proteoglycan link protein1 (Hapln1), G protein-coupled receptor17 (Gpr17), and myelin protein zero (Mpz) (Additional file 1: Supplementary data 2).

In mice treated with IQG5, compared to control, 1758 genes were up-regulated, including lymphocyte antigen 6 complex (Ly6a), phospholipid transfer protein (pltp), lysyl oxidase (Lox), collagen type I alpha1 (Col1a1), and collagen type I alpha2 (Col1a2) (Additional file 1: Supplementary data 1). However, 1017 genes were down-regulated, including nuclear RNA export factor (Nxf3), hyaluronan and proteoglycan link protein1 (Hapln1), and hem binding protein 2 (Hebp2).

In HEG5 group, 1004 genes were up-regulated, including lymphocyte antigen 6 complex (Ly6a), lysyl oxidase (Lox), collagen type I alpha1 (Col1a1), and collagen type I alpha2 (Col1a2). However, 412 genes were down-regulated including hyaluronan and proteoglycan link protein1 (Hapln1). These data suggested that collagen type XII alpha1, was up-regulated while nuclear RNA export factor (Nxf3) was down-regulated by CaG5 treatment, indicating that they might serve as potential therapeutic markers for anti-cancer agent (Additional file 1: Supplementary data 1 and 2).

## Discussion

As known glycosaminoglycans (GAGs), heparin or heparan sulphate glycosaminoglycans are linear polymers (polysaccharides) with above 20 disaccharide units of N-acetylated D-glucosamine α (1–4) linked to glucuronic acid. As used GAGs in the present study, it has been reported that glycosaminoglycans from *C. molossus* and queen of *B. ignitus* mainly contain D-glucosaminic acid as an acid monosaccharide and N-acetyl-galactosaminitol as an amino monosaccharide [8, 9]. Furthermore, they contain α-glucose and D-mannitol as neutral monosaccharides [8, 9] and HEG contains fucose as a neutral monosaccharide.

Glycosaminoglycan was macromolecule polymer. Therefore its precise mechanism of action in vitro study, each N-glycan under molecular weight 3 K Dalton, was preferentially used and characterized. From new GAG supply source, insect N-glycans were identified as polymer with hexose and N-acetylhexose in this study. N-glycans of CaG were identified as Hex_3_, HexNAc_3_dHex_1_. N-glycans of IQG were as Hex_3_, HexNAc_2- 4_dHex_1_, Hex_3_HexNAc_3_ or Hex_3_HexNAc_6_. N-glycans of HEG were as a Hex_7_, Hex_7_HexNAc_6_. Cell growth ratios of melanoma cells were significantly decreased by GAG N-glycan samples (40 μg/ml), especially CaG (70.9% decrease, CaG vs CON, *p* < 0.05), IQG (76.2% decrease) and HEQ (81.3% decrease, HEG vs CON, *p* < 0.01) compared control (Fig.1b). Recently, as various biological activities of GAG or the N- glycan (N-glycan) are known, they use as the medical materials and the value as the restriction resources and functional molecule increases with the specificity being controlled by glycosyltransferases such as trifling change and the delicate difference of the sugar chain. Especially, the role is known that it affects growth and infiltration of the cancer cell while the role being performed as the biological filter and controlling the tensile strength of skin and collagen fiber composition.

Used endogenous inhibitor as an anticancer mechanism clue in this study, TIMP-2 has many distinct properties and functions independent of MMP inhibition, including inhibition of tumor growth and reduction of angiogenesis through decreased endothelial cell proliferation and migration by interacting with alpha3 beta1 (α3β1) integrin on endothelial cells [11]. In the present study, TIMP-2 level in CaG group was significantly increased compared to that in the control group, showing the highest inhibition level among three treated groups.

ECM participates in multiple biological events, including tumor angiogenesis and represents a network of biopolymers, offering a dynamic tissue-specific structure that is responsible for transmitting extracellular signals to cells [16]. There are some reports on adhesion molecule and glycosaminoglycan. For example, collagen I and oxidized glycosaminoglycan (chondroitin sulfate) can improve stability and cell response by intrinsic cross-linking of multilayers [17]. Fibronectin, a fibroblast surface protein, can bind to cell surface integrin and many extracellular matrix (relaxed, healthy ECM and strained, tumor ECM) proteins. It plays important roles in cell adhesion, migration, differentiation, and transformation related to cancer invasion [18]. It has alternatively spliced fibronectin domains. Domain-A and domain-B of fibronectin are expressed during vasculogenesis in the embryo. However, they are essentially undetectable in adults [19]. These domains and tumor vascular antigens are potential therapeutic vaccines by targeting growth of primary tumors [19].

**Fig. 5:**
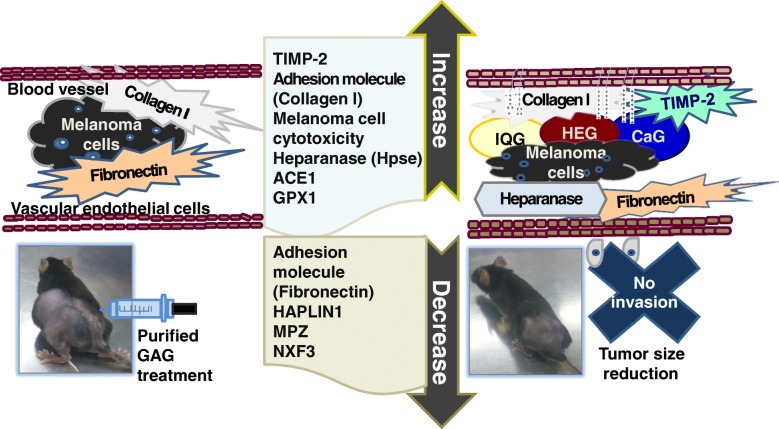
Proposed role of dung beetle/bumblebee queen glycosaminoglycan against melanoma. CaG can strengthen ECM by increasing activity of TIMP-2 and adhesion activity on to collagen 1 to inhibit changes of ECM, leading to tumor cell invasion and progression along with increased activities of heparanase and angiotensin converting enzyme as well as fibronectin regulation (small amount ECM adhesion) in melanoma cells

The adhesion ability of CaG or IQG for ECM proteins was in the following order: collagen I > fibronectin > vibronectin > laminin > tenascin > collagen IV > collagen II > BSA. To search for active compounds as insect crude drugs, dung beetle and queen of *B. ignitus* were studied. Dung beetle, *C. molossus* GAG has been found to possess anti-aging activity to prevent or treat fatty liver or hyperlipidemia with cardio-protection ability [8]. Like other glycosaminoglycan, to prevent cancer procession, interrupting invasion and angiogenesis of cancer cells has been proposed as a strategy [17, 18] (Fig. 5).

In the present *vivo* study, melanoma tumor size reductions were observed in insect glycosaminoglycan-treated groups as following: CaG 22.5% reduction; IQG 34.55% reduction; and HEG 22.55% reduction. However, tumor size was increased as time went by in the CON (PBS) group.

Furthermore, data generated from DNA microarray supported the anti-cancer effect of CaG, IQG, and HEG, showing meaningful gene expression changes in melanoma induced mice at 6th week after treatment. Compared to CON group, CaG5 treated mice group showed upregulation of 192 genes, including Col12a1 involved in cytoskeletal reorganization [20, 21] and Hbb-bt, amino levulinic acid synthase2, erythroid (Alas2) [22, 23], and heparanase (Hpse) [24]. It also showed downregulation of 152 genes, including nuclear RNA export factor (Nxf3) [25], hyaluronan and proteoglycan link protein1 (Hapln1) [26], G protein-coupled receptor17 (Gpr17) [27, 28], and myelin protein zero (Mpz) [29–31]. These data suggest that collagen type XII alpha1 was up-regulated while nuclear RNA export factor (Nxf3) was down-regulated by CaG5 treatment, indicating their potential as therapeutic markers for anti-cancer agents. Because these GAGs could be main mediators of communication (cell–cell and cell–ECM communication) in the intracellular space [32], the use of CaG, IQG or HEG might play important roles in inhibiting malignant transformation and tumor metastasis. These results suggest that CaG, IQG, and HEG could be used as natural anti-cancer agents and functional food, like Sulodexide [33, 34]. Furthermore, these GAGs have low molecular weight N-glycan, like low-molecular weight heparins. They can inhibit melanoma growth and provide survival benefit for melanoma mice [35] through a myriad of mechanisms [36, 37]. Anticoagulant like heparin GAG has been reported to have anticancer activity, contributed anti-angiogenesis [38]. CaG treatment in this experiment, showed that heparanase (degradation enzyme of heparin sulfate), had high expression level in gene expression by quantitative real-time PCR analysis compared to angiotensin converting enzyme [39]. Recently report is also suggested that NK heparanse regulate cancer growth by immune modulation [40]. Throughout these results, we could demonstrate the potential efficacy of insect GAG comprising CaG as an anti-cancer treatment against not only melanoma but also other cancer based on cytotoxicity against cancer cells (CT-26, colon carcinoma cell) and non-cytotoxicity in normal CHO cells with immune modulation [41].

## Conclusions

Glycosaminoglycan (GAG) from dung beetle, IQG, and HEG displayed anti-cancer properties (tumor size reduction and survival time elongation). These glycans hold great promises as anti-cancer agents. Our results also demonstrated the potential value of *C. molossus* glycosaminoglycan in lessening deleterious aspects of B16F10 melanoma, including serum parameters and the gene expression levels in melanoma induced mice after GAG treatment. The inhibition of these insect GAGs could offer another promising approach to treat including melanomas.

## Additional file

Additional file 1: Supplementary Data 1. Up-regulated genes in melanoma tissue of B16F10 melanoma induced mice treated with insect GAG over a 6-week period. Supplementary Data 2. Down-regulated genes in melanoma tissue of B16F10 melanoma induced mice treated with insect GAG over a 6-week period. (DOCX 3436 kb)

## Abbreviations

ACE1: Angiotensin converting enzyme1
ALP: Alkaline phosphatase
CaG: Dung beetle (*C. molossus*) glycosaminoglycan
CaG5: *C. molossus* (dung beetle) glycosaminoglycan 5 mg/kg
CEA: Carcinoembryonic antigen
Colla1: Collagen, type I, alpha1
CON: Control group
CS: Chondroitin sulfate
CS-E: Chondroitin sulfate chains containing E-disaccharide units
DMEM: Dulbecco’s modified Eagle medium
DS: Dermatan sulfate
ECM: Extracellular matrix
GAG: Glycosaminoglycan
GPX1: Glutathione peroxidase1
Hapln1: Hyaluronan and proteoglycan link protein1
HEG: *H. sanguinea* glycosaminoglycan
HeG5: *H. sanguinea* glycosaminoglycan 5 mg/kg
HMVEC: Human microvascular (cardiac) endothelial cells
Hpse: Heparanase
HS: Heparin sulfate
HSPG: Heparan sulfate proteoglycan
ICAM: Intercellular adhesion molecule
IQG: Bumblebee (*B. ignitus*) queen glycosaminoglycan
IQG5: *B. ignitus* queen glycosaminoglycan 5 mg/kg
LDH: Lactate dehydrogenase
MMP: Matrix metalloproteinase
Mpz: Myelin protein zero
Nxf3: Nuclear RNA export factor
PBS: Phosphate buffered saline
RACE: Advanced glycation end product
TIMP-2: Tissue inhibitor metalloprotease
