# Supplementary material for: Anti-cancer effect of dung beetle glycosaminoglycans on melanoma
Source: BMC Cancer. 2019 Jan 5;19:9. doi: 10.1186/s12885-018-5202-z (PMC6321666; doi:10.1186/s12885-018-5202-z)
Supplement: Supplementary file 1 — Supplementary Data 1. Up-regulated genes in melanoma tissue of B16F10 melanoma induced mice treated with insect GAG over a 6-week period. Supplementary Data 2. Down-regulated genes in melanoma tissue of B16F10 melanoma induced mice treated with insect GAG over a 6-week period. (DOCX 3436 kb) [file 12885_2018_5202_MOESM1_ESM.docx]

**Supplementary Data 1** Up-regulated genes in melanoma tissue of B16F10 melanoma induced mice treated with insect GAG over a 6-week period

**Supplementary Data 2** Down-regulated genes in melanoma tissue of B16F10 melanoma induced mice treated with insect GAG over a 6-week period
